# Novel compound heterozygous variants in the *PLD1* gene causing cardiac valve dysplasia 1 complicated with dilated cardiomyopathy: a case report

**DOI:** 10.3389/fcvm.2026.1879157

**Published:** 2026-07-29

**Authors:** Xiaohuan Zhang, Xiang Liu, Wenjing Zhou, Yingnan Liao

**Affiliations:** 1 Genetic Diseases Key Laboratory of Sichuan Province, the Department of Medical Genetics, Sichuan Academy of Medical Sciences & Sichuan Provincial People's Hospital, School of Medicine, University of Electronic Science and Technology of China, Chengdu, Sichuan Province, China; 2 Genetic Diseases Key Laboratory of Sichuan Province, the Department of Laboratory Medicine, Sichuan Academy of Medical Sciences & Sichuan Provincial People's Hospital, School of Medicine, University of Electronic Science and Technology of China, Chengdu, Sichuan Province, China; 3 Department of Pediatrics, Chongqing Health Center for Women and Children, Chongqing, China; 4 Department of Pediatrics, Women and Children’s Hospital of Chongqing Medical University, Chongqing, China

**Keywords:** cardiac valve dysplasia 1 (CVDD1), compound heterozygous variants, dilated cardiomyopathy (DCM), genetic diagnosis, missense variant, PLD1 gene, splice site variant

## Abstract

**Objective:**

Congenital heart disease (CHD), the most common congenital defect, accounts for one-third of all congenital anomalies. Genetic causes are identified in 20%–30% of CHD cases, mostly involving abnormal valve formation. *PLD1* gene variants are postnatally associated with pulmonary stenosis, valvular regurgitation, left ventricular dilation and right ventricular hypoplasia.

**Methods:**

Clinical data of the proband were collected, and targeted capture high-throughput sequencing was performed on the proband and his family members. The detected gene variants were interpreted according to the ACMG guidelines, and the correlation between variants and diseases was analyzed by combining clinical phenotypes and genetic characteristics.

**Results:**

The proband presented with recurrent fatigue, palpitations, and aggravated symptoms for 2 days, with a history of cardiac valve disease for more than 17 years and three open-heart surgeries. Echocardiography showed left ventricular enlargement, decreased ventricular wall motion amplitude, and mild tricuspid regurgitation. Genetic testing identified two novel compound heterozygous variants in the *PLD1* gene: NM_002662.5: c.434 + 1G > T (a splice site variant, inherited from the father, classified as pathogenic) and NM_002662.5:c.2681A > G (a missense variant, NP_ 002653.1: p.Tyr894Cys, inherited from the mother, classified as a variant of uncertain significance).

**Conclusion:**

These two novel compound heterozygous variants in *PLD1* may be the pathogenic genetic cause of cardiac valve dysplasia 1 (CVDD1) complicated with dilated cardiomyopathy (DCM) in this patient, which expands the variant spectrum of *PLD1* and provides valuable evidence for genetic diagnosis and genetic counseling of CVDD1 and related cardiomyopathies.

## Introduction

Congenital heart disease (CHD) refers to structural and functional abnormalities of the heart and great intrathoracic vessels caused by abnormal embryonic development. According to the International Paediatric and Congenital Cardiac Code (IPCCC) and the Eleventh Revision of the International Classification of Diseases (ICD-11), CHD is classified as a group of congenital anomalies affecting the heart and great vessels, with standardized diagnostic codes for each specific structural lesion (1–4) As the most common birth defect in China, CHD has an incidence rate of 8.98‰ (5). The etiology of CHD is complex, and most cases result from the combined effects of environmental and genetic factors (6, 7). Studies have reported that genetic factors are implicated in approximately one-third of CHD cases, among which chromosomal numerical abnormalities account for about 10%, chromosomal structural aberrations for 5% to 20%, and monogenic variants for approximately 10% (8, 9). Within the IPCCC/ICD-11 framework, congenital cardiac valvular dysplasia is classified under the category of “ Congenital anomaly of an atrioventricular valve or atrioventricular septum” (3). This group of disorders is characterized by cardiac valvular malformations accompanied by congenital cardiac structural abnormalities, and is further classified into three subtypes based on the underlying genetic cause: X-linked cardiac valvular dysplasia (CVDDX, FLNA), cardiac valvular dysplasia 1 (CVDD1, *PLD1*), and cardiac valvular dysplasia 2 (CVDD2, ADAMTS19) (10). CVDD1 is an autosomal recessive genetic disease caused by biallelic variants of *PLD1* (11–14), which mainly affects the right heart valves and is featured by more complex cardiac structural malformations, more severe phenotypes and poorer prognosis. Phospholipase D (PLD) is an essential signaling enzyme in mammalian cells, which generates phosphatidic acid (PA) by hydrolyzing phosphatidylcholine (PC) upon the activation of various cell surface receptors. PLD1 and PLD2 are the most extensively studied PLD isoforms (15). The *PLD1* gene is located on chromosome 3q26.31, consisting of 27 exons and encoding a 1074-amino acid protein (14). *PLD1* is highly expressed in the pancreas and heart, and participates in the key steps of numerous cellular pathways, including signal transduction, membrane trafficking and the regulation of mitosis (15, 16).

Dilated cardiomyopathy (DCM) is a heterogeneous cardiomyopathy characterized by left ventricular/biventricular enlargement and impaired systolic function, with genetic factors accounting for approximately 30%–50% of sporadic cases. Although *PLD1* variants have been identified as the genetic cause of CVDD1, and the co-occurrence of CVDD1 and DCM in *PLD1* variant carriers has been reported in limited case reports to date (17), the genotype-phenotype correlation between specific *PLD1* variants and this combined phenotype remains largely unelucidated. Notably, novel compound heterozygous variants in *PLD1* underlying CVDD1 complicated with DCM have not been reported previously, and the potential pathogenic mechanisms by which *PLD1* variants drive both congenital valve dysplasia and progressive cardiomyopathy are still poorly understood. Herein, we report a 16-year-old male patient with CVDD1 complicated with DCM caused by two unreported compound heterozygous variants in *PLD1*, and analyze the clinical and genetic characteristics of this case to provide new insights into the *PLD1*-related cardiac disease spectrum and its genotype-phenotype associations.

## Patient information

A 16-year-old male (II-1 in the pedigree; Figure 2A) was admitted to our institution with a 4-month history of recurrent fatigue and palpitations, with symptom exacerbation over the 2 days preceding admission. Each episode occurred without identifiable triggers, lasted approximately 30 min, and resolved spontaneously with rest. The patient's medical history is significant for severe mitral regurgitation diagnosed neonatally, necessitating multiple surgical procedures throughout childhood. He has no family history of congenital heart disease or cardiomyopathy (Figure 2A).

## Timeline

The key clinical events and interventions are summarized in Table 1.

**Table 1:** Timeline of key clinical events and interventions in the proband.

Age	Event	Key findings/outcome
Birth	Congenital cardiac valve abnormalities identified	Congenital mitral valve dysplasia; severe mitral regurgitation
10 months	First mitral valvuloplasty	Postoperative EF = 49%; mild mitral regurgitation
2 years	Second mitral valvuloplasty (for mitral valve prolapse)	Postoperative EF = 62%; severe mitral regurgitation persisted
7 years	Mechanical mitral valve replacement (3rd open-heart surgery)	Postoperative successful valve replacement
16 years (current admission)	Admission for recurrent fatigue and palpitations	EF = 37%; left atrial and ventricular enlargement

## Diagnostic assessment

### Physical examination on admission

The patient was alert and oriented. Blood pressure was 121/60 mmHg, heart rate was 84 bpm, respiratory rate was 18 breaths/min, and body temperature was 36.5 °C. Cardiac auscultation revealed a metallic prosthetic valve click, with a grade III systolic murmur best heard at the apex. No diastolic murmur, gallop, or pericardial friction rub was detected. Jugular venous pressure was slightly elevated, with negative hepatojugular reflux. Lung auscultation revealed coarse breath sounds bilaterally without crackles. No peripheral edema was present. Abdominal examination was unremarkable.

### Echocardiography

**Figure 1:**
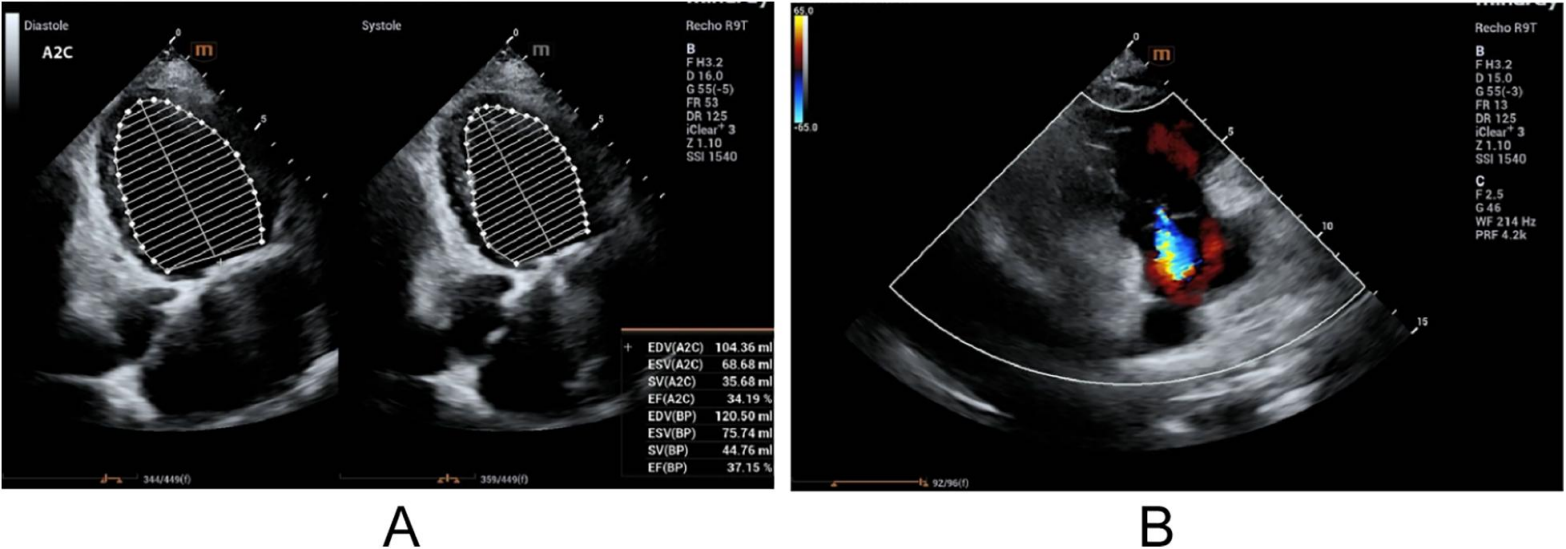
Echocardiographic assessment of left ventricular function and valvular regurgitation. **(A)** Two-chamber (A2C) views with speckle-tracking strain analysis for evaluation of myocardial deformation. **(B)** Corresponding color Doppler image demonstrating regurgitant flow across the cardiac valve.

(Figures 1A,B) revealed the following findings:

### Structural abnormalities

Left atrial and left ventricular enlargement (LA = 52 mm, LVEDd = 63 mm), with mildly enlarged right ventricle (basal transverse diameter ≈ 45 mm). Interventricular septal and left ventricular posterior wall thicknesses were within normal limits (9 mm each). Left ventricular wall motion amplitude was decreased. The inferior vena cava was dilated (21 mm) with a respiratory variation rate < 50%, suggesting elevated right atrial pressure.

### Prosthetic valve assessment

The mechanical mitral valve prosthesis demonstrated a stable frame, normal leaflet movement, effective orifice area of ≈ 1.86 cm^2^, and a mean transvalvular pressure gradient of ≈ 4.4 mmHg, indicating normal prosthetic valve function.

### Valvular regurgitation

Grade I tricuspid regurgitation (TRG = 25.2 mmHg) was noted. No abnormal blood flow was detected across the other valves.

Left ventricular systolic function was moderately reduced (Simpson EF = 37%), and diastolic function was impaired (MV E/e′ ratio = 31.17).

*Cardiac computed tomography (CT)* confirmed cardiac enlargement, with an annular high-density shadow in the mitral valve area consistent with the mechanical valve prosthesis. Sternal postoperative changes were consistent with multiple prior open-heart surgeries.

### Diagnostic reasoning

The patient's presentation involves congenital mitral valve dysplasia (supported by infantile severe regurgitation, failed valvuloplasties, and valve replacement, consistent with CVDD1 due to biallelic PLD1 variants) and dilated cardiomyopathy (LVEDd 63 mm, EF 37%, with progressive decline from 62% at age 2 despite successful valve replacement at age 9, suggesting intrinsic myocardial involvement beyond hemodynamic stress). Compound heterozygous *PLD1* variants provide molecular confirmation.

### Differential diagnosis

Hemodynamic DCM from chronic valve disease is unlikely as the sole cause given normal prosthetic function. Myocarditis is excluded by no acute illness or enzyme elevation and the chronic course. Isolated idiopathic DCM is less likely given congenital valve disease and *PLD1* variants. Tachycardia-induced cardiomyopathy is ruled out by no documented arrhythmias.

### Prognostic characteristics

EF 37% at age 16 increases risks of heart failure, arrhythmias, and sudden death, warranting ICD evaluation. Biallelic *PLD1* variants may indicate a more aggressive course. Young age allows consideration of transplantation if progression continues.

### Clinical Diagnosis

(1) Congenital mitral valve dysplasia (IPCCC: 06.02.11; ICD-11: LA87.1) with secondary dilated cardiomyopathy, consistent with cardiac valve dysplasia 1 (CVDD1, OMIM: 212093); (2) Left ventricular enlargement with moderately reduced systolic function (Simpson EF = 37%); (3) Mild tricuspid regurgitation (IPCCC: 06.01.25; ICD-11: LA87.0); (4) Acute decompensated heart failure (EF reduction type), NYHA class II–III; (5) Status post mechanical mitral valve replacement (3rd open-heart surgery).

## Therapeutic intervention

### Surgical intervention

The patient underwent three open-heart surgeries, beginning at approximately one year of age for congenital mitral valve dysplasia. The final surgery, performed at approximately seven years of age, involved mechanical mitral valve replacement. Postoperatively, lifelong anticoagulation with warfarin was initiated.

### Pharmacological intervention for heart failure

At the most recent admission (age 16 years) for acute decompensated heart failure (NYHA class II–III), the patient was initially treated with levosimendan for inotropic support, as conventional inotropic and vasodilator therapy had proven insufficient. Intravenous furosemide (10 mg daily) was administered for volume management. Guideline-directed medical therapy (GDMT) for heart failure with reduced ejection fraction was subsequently initiated and optimized prior to discharge, comprising sacubitril/valsartan (50 mg twice daily), bisoprolol (1.25 mg once daily), dapagliflozin (10 mg once daily), spironolactone (20 mg once daily), and vericiguat (2.5 mg once daily).

### Anticoagulation

Warfarin (2.5 mg once daily) was continued for the mechanical mitral prosthesis, with a target INR of 2.0–3.0.

## Follow-up and outcomes

At the time of this report, the patient's clinical status was characterized by progressive DCM (EF = 37%) secondary to long-standing CVDD1, despite three surgical interventions. Transthoracic echocardiography confirmed stable mechanical mitral valve prosthesis function, with a mean transprosthetic gradient of 4.4 mmHg and an effective orifice area of 1.86 cm^2^, without perivalvular leakage or thrombus. Left atrial and ventricular enlargement persisted, with moderately reduced left ventricular systolic function and grade I tricuspid regurgitation. Iron deficiency was identified incidentally (transferrin saturation 18.2%, serum iron 12.9 μmol/L), for which supplementation was recommended. Genetic testing identified compound heterozygous variants in *PLD1* (detailed in the Results section). The patient's parents, who are heterozygous carriers of the respective *PLD1* variants, were clinically asymptomatic at the time of evaluation. Longitudinal cardiac monitoring, including regular echocardiography and functional assessment, was recommended for both carrier parents.

## Materials and methods

### Sample collection

Genomic DNA was extracted from peripheral blood leukocytes (2 mL) of the proband and his parents using the standard phenol-chloroform method, and the DNA concentration and purity were detected by a NanoDrop 2000 spectrophotometer (Thermo Fisher Scientific, USA).

### Genetic testing

Targeted capture high-throughput sequencing was performed using the Human Cardiac Disease Gene Capture Kit (Agilent Technologies, USA), and sequencing was conducted on the Illumina NovaSeq 6000 platform (Illumina, USA) with a sequencing depth of ≥100×.

### Nomenclature and classification of cardiac diagnoses

Cardiac structural abnormalities and clinical diagnoses were classified according to the International Paediatric and Congenital Cardiac Code (IPCCC) and the 11th Revision of the International Classification of Diseases (ICD-11). The IPCCC provides a globally standardized nomenclature for congenital and paediatric cardiac diseases, ensuring consistent diagnostic classification across clinical and research settings (3).

### Variant analysis

The sequencing data were compared with the human reference genome (GRCh37/hg19), and variant calling was performed using GATK software. The detected variants were annotated by databases such as dbSNP, 1000 Genomes Project, ExAC, and ClinVar. The pathogenicity of variants was evaluated according to the guidelines of the American College of Medical Genetics and Genomics (ACMG) (18). Sanger sequencing was used to verify the identified *PLD1* variants, with primers designed by Primer3 (v0.4.0) (primer sequences are available upon request). Sequencing was performed on an ABI 3730xl DNA Analyzer (Applied Biosystems, USA), and data were analyzed with Chromas (v2.6.6). Variant nomenclature follows the HGVS guidelines (version 21.1.0). The MANE Select transcript NM_002662.5 was used as the reference sequence for *PLD1* variant reporting.

## Results

**Figure 2:**
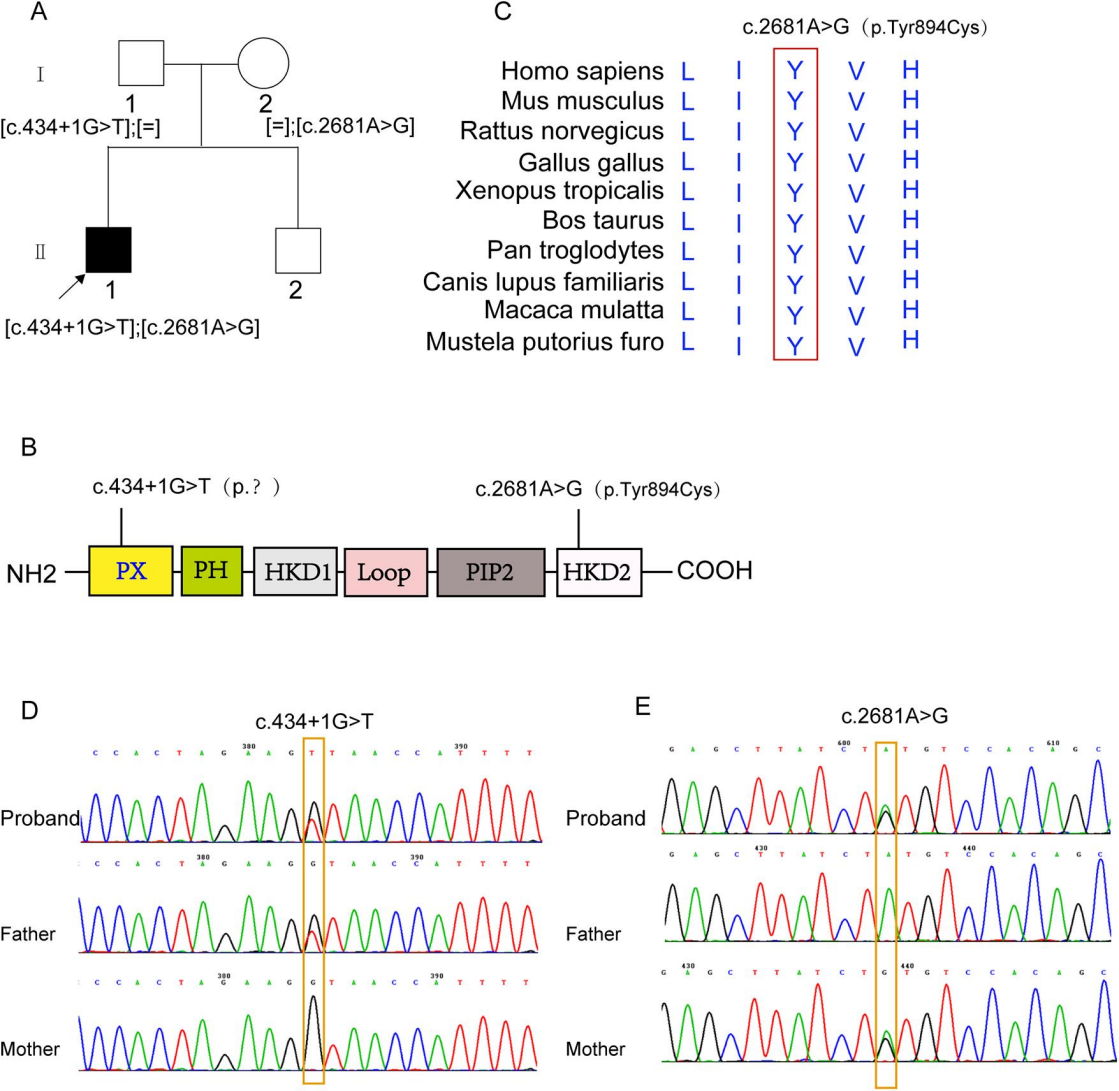
Genetic analysis of the *PLD1* compound heterozygous variants in the proband's family. **(A)** Pedigree of the non-consanguineous family. The arrow indicates the proband (II-1). Genotypes are shown below each individual using HGVS nomenclature based on the MANE Select transcript NM_002662.5: the proband carries [c.434 + 1G > T];[c.2681A > G] (compound heterozygous); the father (I-1) carries [c.434 + 1G > T];[ = ]; the mother (I-2) carries [ = ];[c.2681A > G]. [ = ] denotes the reference allele. II-2 represents the proband's younger brother, who is unaffected and has not undergone genetic testing. **(B)** Schematic of PLD1 protein domains with variant locations (black arrows). NH2, amino terminus; COOH, carboxyl terminus; PX, PH, HKD1, Loop, PIP2, HKD2: functional domains. c.434 + 1G > T (p.?) is predicted to disrupt splicing; c.2681A > G (p.Tyr894Cys) is a missense variant in the HKD2 domain. **(C)** Evolutionary conservation analysis of the p.Tyr894Cys residue across 10 vertebrate species (red box: the conserved Tyr894). **(D)** Sanger sequencing chromatograms for c.434 + 1G > T: proband (heterozygous G/T), father (heterozygous G/T), mother (wild-type G/G). **(E)** Sanger sequencing chromatograms for c.2681A > G: proband (heterozygous A/G), mother (heterozygous A/G), father (wild-type A/A).

Targeted capture high-throughput sequencing identified compound heterozygous variants in the *PLD1* gene of the proband, constituting a compound heterozygous state: NM_002662.5 (*PLD1*):[c.434 + 1G > T];[c.2681A > G]. The two variants are described individually below: NM_002662.5: c.434 + 1G > T (a splice site variant): This splice site variant has not been previously reported in the literature. In population databases (gnomAD v2.1.1), this variant is absent, meeting the criterion PM2_Supporting. According to the ACMG/AMP guidelines (19, 20), splice site variants affecting the conserved +1 donor site are classified as pathogenic strong evidence (PVS1). Family segregation analysis by Sanger sequencing confirmed that the proband's father carried this variant, while the mother did not. This variant was classified as pathogenic (PVS1 + PM2_Supporting + PP4). NM_002662.5: c.2681A > G (NP_002653.1:p.Tyr894Cys, a missense variant): This is a missense variant resulting in the substitution of tyrosine at position 894 with cysteine. This missense variant (p.Tyr894Cys) has not been previously reported in the literature. In population databases (gnomAD v2.1.1), the allele frequency of this variant is 0.00001596, below the threshold for PM2_Supporting (allele frequency < 0.001 in gnomAD). A pathogenic variant was identified in trans (PM3). Family segregation analysis confirmed that the proband's mother carried this variant, while the father did not. This variant was classified as a variant of uncertain significance (PM3 + PM2_Supporting + PP4). PP4 evidence was based on the proband's clinical phenotype (CVDD1 complicated with DCM) which was highly consistent with the known clinical manifestations of *PLD1*-related cardiac diseases. Sanger sequencing verified that the two variants of the proband were inherited from his father and mother respectively, forming a compound heterozygous state (Figures 2A–E). The pedigree chart of the non-consanguineous family showed no other affected members (Figure 2A).

## Discussion

Congenital heart disease (CHD) is one of the most common birth defects in China, with an incidence rate of 8.98‰. The etiology of CHD is predominantly multifactorial, resulting from complex interactions between genetic and environmental factors (21, 22). Genetic contributors are identified in approximately one-third of cases, encompassing chromosomal abnormalities, copy number variants, and monogenic disorders (23); however, the majority of CHD cases cannot be attributed to a single genetic cause. Large-scale exome analyses have estimated that rare damaging recessive genotypes contribute to at least 2.2% of CHD cases (24). Congenital cardiac valvular dysplasia represents a specific subset of CHD with a stronger monogenic contribution, particularly involving genes such as *FLNA* (10, 25)*, PLD1,* and *ADAMTS19* (26). Cardiac valvular dysplasia 1 (CVDD1, OMIM: 212093), an autosomal recessive disorder caused by biallelic *PLD1* variants, exemplifies a monogenic form of valvular CHD (22). To date, the co-occurrence of CVDD1 and dilated cardiomyopathy (DCM) in *PLD1* variant carriers has been reported in limited case reports worldwide, and the genotype-phenotype correlation and underlying pathogenic mechanisms remain largely unelucidated. This study identified two novel compound heterozygous variants (NM_002662.5:[c.434 + 1G > T];[c.2681A > G]) in a 16-year-old male patient with long-standing CVDD1 and progressive DCM, which enriches the variant spectrum of *PLD1* and provides new evidence for the association between *PLD1* variants and combined cardiac valve and myocardial damage.

The link between *PLD1* variants and cardiac valvular defects was first reported in 2017 (13), and subsequent studies have clarified its stage-specific expression during cardiac development and core role in valvulogenesis: PLD1 expression is spatiotemporally restricted in rat and chick embryonic hearts (27), consistent with cardiac apoptotic dynamics and its pro-apoptotic function, and *PLD1*-knockout mice exhibit typical right heart valve malformations (tricuspid regurgitation, pulmonary valve stenosis/thickening) that mimic human CVDD1 phenotypes (13). As a key signal-transducing enzyme, PLD1 hydrolyzes phosphatidylcholine to generate the lipid second messenger phosphatidic acid (PA) (11), whose enzymatic activity is closely associated with severe congenital cardiac valvular defects. PA modulates multiple cardiac functions, including regulating endocardial and endoplasmic reticulum Ca^2^⁺ transport, elevating intracellular free Ca^2^⁺ in cardiomyocytes, enhancing cardiac contractility, and acting as a signal sensor for cardiac hypertrophy (28)—laying the molecular foundation for *PLD1*-mediated cardiac development and functional regulation. Moreover, *PLD1* is confirmed to be essential for endothelial-mesenchymal transition (EndoMT), a critical early step in embryonic valvulogenesis; *PLD1* inhibition significantly reduces EndoMT in chick embryo atrioventricular cushion (AVC) explants (11), directly linking *PLD1* loss-of-function to abnormal valve formation.

Since the first identification of *PLD1* variants in severe valvular malformation patients by Ta-Shma et al. (13) successive studies have expanded the variant and phenotype spectrum of *PLD1*-related CHD (Table 2). Qiao et al. (29) identified two novel missense variants (*PLD1* c.2447G > C/p.Arg816Pro, c.1171C > T/p.Arg391Cys) in fetal CHD cohorts and confirmed they reduce *PLD1* protein stability. A landmark 2021 study by Lahrouchi et al. (11) constructed an international cohort of 30 patients with biallelic *PLD1* variants, reporting 31 variants (predominantly missense and protein-truncating) that cluster in the conserved HxKxxxD (HKD) domain and C-terminal region essential for enzymatic activity (30)—distinct from benign variants scattered across the protein. This study also first confirmed that recessive *PLD1* variants can cause neonatal heart disease without congenital cardiac defects. In a whole-exome study of 5,424 CHD probands, recessive genotypes in *PLD1* were identified, with founder variants accounting for 74% of the recessive contribution among Ashkenazi Jewish probands (24). Subsequent studies identified novel pathogenic *PLD1* variants in clinical cases: Cai et al. (2023) (31) found a compound heterozygous variant (c.1132dupA/p.I378fs + c.1171C > T/p.R391C) in the HKD1 domain of a fetal CHD case, with c.1132dupA classified as pathogenic by ACMG guidelines; Zhu et al. (2024) (14) validated that the splice variant c.1062-59A > G causes intron retention and exon skipping, leading to complete *PLD1* loss-of-function and structural atrioventricular valve defects. Collectively, these studies confirm *PLD1* as a core pathogenic gene for congenital cardiac valvular dysplasia, with its pathogenic variants mainly disrupting enzymatic activity and EndoMT, and inheriting in an autosomal recessive pattern.

**Table 2:** *PLD1* variants reported in the literature and their clinical characteristics.

Study (year)	Reference	*PLD1* variant	Variant type	Genotypic characteristics	Clinical phenotype	Cardiomyopathy (Y/N)	Treatment	ACMG classification
Ta-Shma et al. (2016) (13)	PMID:27799408	c.1325A>C(p.His442Pro)	Missense mutation	Homozygous	A-II-3：Newborn: severe pulmonary stenosis, moderate RV hypoplasia, 3 medium VSDs, large ASD, no extracardiac symptoms 22y: central cyanosis, haematocrit 72%; A-II-4: Newborn: severe cyanosis, tricuspid/pulmonary atresia, single left ventricle, small pulmonary arteries, no extracardiac symptoms 16y: central cyanosis	N	A-II-3：3y: palliative Blalock-Taussig shunt 4.5y: bidirectional Glenn operation 22y: one-and-half ventricular repair; A-II-4：2w: palliative Blalock-Taussig shunt 11m: bidirectional Glenn operation 16y: Fontan operation	Likely pathogenic (PS3 + PM2_Supporting + PP3 + PP4)
		c.1484_1485del(p.Thr495fs32*)	Frameshift mutation	Homozygous	Birth: right eye cataract, no other abnormalities 5m: fever, diarrhoea, severe mitral regurgitation (MR) Progressive: poor thriving, left atrial/ventricular dilation, worsened MR	N	9m: mitral valve repair (for anterior leaflet prolapse due to chordae elongation)	Pathogenic (PVS1 + PM2_Supporting + PP4)
		c.2882 + 2T>C/c.1484_1485del(p.Thr495fs32*)	Splicing mutation/Frameshift mutation	Compound heterozygous	Birth: mild cyanosis, mild tricuspid/pulmonic stenosis, mild RV hypoplasia, mild MR, right-to-left shunt (patent foramen ovale) 1m: tachypnoea, moderate-to-severe MR, left atrial dilation	N	No surgeries Asymptomatic at 17 years of age	Pathogenic (PVS1 + PM2_Supporting + PP4)
Qiao et al. (2020) (29)	PMID:33142350	c.2447G>C (p.Arg816Pro)/c.1171C>T (p.Arg391Cys)	Missense mutation	Compound heterozygous	Left ventricular endocardial fibroelastosis, aortic atresia	Y	Unreported (Detected in fetal period)	Variant of uncertain significance (PM2_Supporting)/Variant of uncertain significance (PM2_Supporting)
Lahrouchi et al. (2021) (11)	PMID:33645542	c.2083C>T (p.Arg695Cys)	Missense mutation	Homozygous	Tricuspid valve defect, pulmonary valve defect, hypoplastic right ventricle	N	Blalock-Taussig shunt	Variant of uncertain significance (PM2_Supporting + PP4)
		c.2083C>T (p.Arg695Cys)	Missense mutation	Homozygous	Tricuspid valve defect, pulmonary valve defect, pulmonary artery hypoplasia	N	Termination of pregnancy (TOP)	Variant of uncertain significance (PM2_Supporting + PP4)
		c.2729G>T (p.Gly910Val)/c.2476G>A (p.Gly826Arg)	Missense mutation	Compound heterozygous	Tricuspid valve defect, pulmonary valve defect, pulmonary artery hypoplasia	N	Blalock-Taussig shunt, bidirectional Glenn procedure, ventricular septal defect, and atrial septal defect closure, Fontan	Variant of uncertain significance (PM2_Supporting + PP3_Moderate + PP4)/Variant of uncertain significance (PM2_Supporting + PP4)
		c.16G>A (p.Glu6Lys)/c.2134C>T (p.Arg712Trp)	Missense mutation	Compound heterozygous	Pulmonary valve defect	N	NA	Benign (BS1 + BP4)/Variant of uncertain significance (PM2_Supporting + PP4)
		c.1085T>C (p.Phe362Ser)/c.1171C>T (p.Arg391Cys)	Missense mutation	Compound heterozygous	Tricuspid valve defect, pulmonary valve defect, hypoplastic right ventricle,pulmonary artery hypoplasia	N	TOP	Variant of uncertain significance (PM2_Supporting + PP3 + PP4)/Variant of uncertain significance (PM2_Supporting + PP4)
		c.1391A>G (p.His464Arg)/c.3000+2T>A(p.Val962Phefs11*)	Missense mutation/Frameshift mutation	Compound heterozygous	Tricuspid valve defect, pulmonary valve defect	N	TOP	Variant of uncertain significance (PM2_Supporting + PP3 + PP4)/Variant of uncertain significance (PVS1_Moderate + PM2_Supporting + PP4)
		c.3142C>G (p.Pro1048Ala)/intragenic_duplication	Missense mutation/intragenic_duplication	Compound heterozygous	Tricuspid valve defect, pulmonary valve defect, hypoplastic right ventricle	Y	Bidirectional Glenn procedure, atrial septectomy and ligation of persistent ductus arteriosus (6 mo)	Variant of uncertain significance (PM2_Supporting + PP3 + PP4)
		c.2002A>T (p.Ile668Phe)	Missense mutation	Homozygous	Tricuspid valve defect, pulmonary valve defect, hypoplastic right ventricle,pulmonary artery hypoplasia	NA	Blalock-Taussig shunt and Bidirectional Glenn procedure awaiting Fontan total cavopulmonary connection procedure	Benign (BS1 + PP4)
		c.2452T>C (p.Tyr818His)	Missense mutation	Homozygous	Tricuspid valve defect	N	NA	Variant of uncertain significance (PP4)
		c.2023C>T (p.Arg675Trp)/c.2098C>T (p.Arg700Cys)	Missense mutation	Compound heterozygous	Tricuspid valve defect, pulmonary valve defect, hypoplastic right ventricle,pulmonary artery hypoplasia	N	NA	Variant of uncertain significance (PM3 + PM2_Supporting + PP4)/Variant of uncertain significance (PM2_Supporting + PP3_Moderate + PP4)
		c.709G>T (p.Gly237Cys)/c.868G>C (p.Glu290Gln)	Missense mutation	Compound heterozygous	Mitral valve defect	N	NA	Variant of uncertain significance (PP3_Moderate + BS1)/Benign (BS1 + BP4)
		c.1325A>C (p.His442Pro)	Missense mutation	Homozygous	Tricuspid valve defect, pulmonary valve defect, hypoplastic right ventricle	N	Central shunt (3 yr), bidirectional Glenn procedure (5 yr), ventricular septal defect closure, 1.5 ventricle repair (22 yr)	Variant of uncertain significance (PM2_Supporting + PP3 + PP4)
		c.1325A>C(p.His442Pro)	Missense mutation	Homozygous	Tricuspid valve defect, pulmonary valve defect, hypoplastic right ventricle,pulmonary artery hypoplasia	N	Blalock-Taussig shunt (2 wk), bidirectional Glenn procedure (1 yr), Fontan (16 yr)	Variant of uncertain significance (PM2_Supporting + PP3 + PP4)
		c.1482_1483del(p.Thr495Argfs*32/c.2882+2T>C	Frameshift mutation/Splicing mutation	Compound heterozygous	Tricuspid valve defect,mitral valve defect	N	Mitral valve repair (8 mo)	Pathogenic (PVS1 + PM2_Supporting + PP4)/Pathogenic (PVS1 + PM2_Supporting + PP4)
		c.1482_1483del(p.Thr495Argfs*32/c.2882+2T>C	Frameshift mutation/Splicing mutation	Compound heterozygous	Tricuspid valve defect	N	-	Pathogenic (PVS1 + PM2_Supporting + PP4)/Pathogenic (PVS1 + PM2_Supporting + PP4)
		c.2098C>T (p.Arg700Cys)/c.2681A> C(p.Tyr894Ser)	Missense mutation/Missense mutation	Compound heterozygous	Tricuspid valve defect, pulmonary valve defect, hypoplastic right ventricle	N	-	Variant of uncertain significance (PM2_Supporting + PP3_Moderate + PP4)/Variant of uncertain significance (PM3 + PP1 + PP4)
		c.1062G>A (p.Trp354*)/c.2681A>C(p.Tyr894Ser)	Nonsense mutation/Missense mutation	Compound heterozygous	Tricuspid valve defect, pulmonary valve defect, hypoplastic right ventricle	N	-	Pathogenic (PVS1 + PM2_Supporting + PP4)/Variant of uncertain significance (PM3 + PP1 + PP4)
		c.892C>T (p.Arg298*)/c.2415delC(p.Leu806fs*0)	Nonsense mutation/Frameshift mutation	Compound heterozygous	Tricuspid valve defect, pulmonary valve defect, hypoplastic right ventricle,pulmonary artery hypoplasia	N	TOP	Pathogenic (PVS1 + PM2_Supporting + PP4)/Pathogenic (PVS1 + PM2_Supporting + PP4)
		c.1219C>T (p.Arg407*)/c.2338G>A (p.Glu780Lys)	Nonsense mutation/Missense mutation	Compound heterozygous	Tricuspid valve defect, pulmonary valve defect, hypoplastic right ventricle,pulmonary artery hypoplasia	N	Cardiac transplantation	Pathogenic (PVS1 + PM2_Supporting + PP4)/Variant of uncertain significance (PM2_Supporting + PP3 + PP4)
		c.2542A>T (p.Arg848*)/c.2191A>T (p.Arg731*)	Nonsense mutation/Nonsense mutation	Compound heterozygous	-	DCM	-	Pathogenic (PVS1 + PM2_Supporting + PP4)/Pathogenic (PVS1 + PM2_Supporting + PP4)
		c.1403T>A (p.Val468Asp)/c.2024G>A (p.Arg675Gln)	Missense mutation/Missense mutation	Compound heterozygous	-	Histiocytoid CMP	-	Variant of uncertain significance (PM2_Supporting + PP4)/Variant of uncertain significance (PM2_Supporting + PP3 + PP4)
Cai et al. (2023) (31)	PMID:36923242	c.1132dupA (p.Ile378*)/c.1171C>T (p.Arg391Cys)	Frameshift mutation/Missense mutation	Compound heterozygous	Fetal right heart dysplasia syndrome. Thickening of tricuspid valve and pulmonary valve leaflets (no obvious opening and closing movement)	N	TOP	Pathogenic (PVS1 + PM2 + PP3 + PP4)/Likely pathogenic(PS4 + PM2 + PP3 + PP4)
		c.1132dupA (p.Ile378*)/c.1171C>T (p.Arg391Cys)	Frameshift mutation/Missense mutation	Compound heterozygous	Pulmonary valve atresia with intact ventricular septum, tricuspid valve defect, foramen ovale and atrial septal defect, aortic arch-pulmonary artery collateral circulation patent ductus arteriosus	N	Deceased	Pathogenic (PVS1 + PM2 + PP3 + PP4)/Likely pathogenic(PS4 + PM2 + PP3 + PP4)
		c.1132dupA (p.Ile378*)/c.1171C>T (p.Arg391Cys)	Frameshift mutation/Missense mutation	Compound heterozygous	Pulmonary valve atresia, tricuspid valve defect, atrial septal defect, arterial catheter reverse blood supply	N	TOP	Pathogenic (PVS1 + PM2 + PP3 + PP4)/Likely pathogenic(PS4 + PM2 + PP3 + PP4)
Wang et al. (2024) (17)	PMID:39681445	c.472C>T(p.Arg158*)	Nonsense mutation	Homozygous	Cardiac valvular dysplasia type 1, left ventricular enlargement with systolic/diastolic dysfunction, mitral valve prolapse with mild regurgitation, tricuspid mild regurgitation, dilated cardiomyopathy with excessive left ventricular trabeculation	Y	NA	Pathogenic (PVS1 + PM3_Supporting + PM2_Supporting)
Zhu et al. (2024) (14)	PMID:39553471	c.1937G>C(p.Gly646Ala) /c.1062-59A>G	Missense mutation/Splicing mutation	Compound heterozygous	Right absent radius, ventricular septal defect	N	TOP	Variant of uncertain significance (PM2_Supporting + PP4)/Variant of uncertain significance (PM2_Supporting + PP4)
		c.1937G>C(p.Gly646Ala) /c.1062-59A>G	Missense mutation/Splicing mutation	Compound heterozygous	Pulmonary atresia, regurgitation and tricuspid valve dysplasia	N	TOP	Variant of uncertain significance (PM2_Supporting + PP4)/Variant of uncertain significance (PM2_Supporting + PP4)
Clinical Case (2026)		c.434+1G>T/c.2681A>G(p.Tyr894Cys)	Splicing mutation/Missense mutation	Compound heterozygous	Dilated cardiomyopathy, left heart enlargement with moderate systolic dysfunction, mild tricuspid regurgitation, acute decompensated heart failure; 17 + years history of congenital cardiac valvular disease	Y	2 times of mitral valvuloplasty, mechanical valve replacement (9 + years ago); anti-heart failure symptomatic treatment	Pathogenic (PVS1 + PM2_Supporting + PP4)/Variant of uncertain significance (PM3 + PM2_Supporting + PP4)

The two *PLD1* variants identified in this patient exhibited distinct pathogenic characteristics and inheritance patterns, and their compound heterozygosity is likely associated with the severe and progressive clinical phenotype. The c.434 + 1G > T splice site variant is likely to disrupt the canonical splice donor site, leading to abnormal pre-mRNA splicing (e.g., exon skipping, intron retention) and the production of a truncated or non-functional PLD1 protein. The p.Tyr894Cys missense variant is located in the conserved functional domain of *PLD1* (Figure 2B), and the substitution of tyrosine (a polar aromatic amino acid) with cysteine (a polar sulfur-containing amino acid) may alter the protein's spatial structure, reduce its phospholipase activity, and further impair cell signal transduction and membrane trafficking in cardiac valve endothelial cells and cardiomyocytes—key processes for normal valve development and myocardial contractility. Notably, the compound heterozygous state of these two rare variants is consistent with the autosomal recessive inheritance pattern of CVDD1. The clinical course of this patient — early-onset valvular malformations, multiple cardiac surgeries, and subsequent progression to DCM—is consistent with the expected phenotypic consequence of biallelic pathogenic variants in an autosomal recessive disorder. However, without functional studies to demonstrate that these specific variants impair PLD1 function, the mechanistic link between the compound heterozygous genotype and the observed phenotype remains presumptive. Regarding the variant classification, it should be noted that although p.Tyr894Cys is currently classified as a VUS according to the ACMG/AMP guidelines, several lines of evidence suggest pathogenic potential. The variant is present in trans with a confirmed pathogenic splice variant (PM3), absent from population databases at appreciable frequency (PM2_Supporting), and the patient's phenotype is consistent with *PLD1*-related cardiac disease (PP4). However, under the point-based scoring framework (19), the combined evidence (4 points) does not reach the threshold for Likely Pathogenic (≥6 points). PP1 could not be applied, as only one affected individual is available in this pedigree. Reclassification may be warranted upon acquisition of additional evidence, such as concordant in silico predictions (PP3), functional validation demonstrating impaired PLD1 enzymatic activity, or identification of additional unrelated affected individuals carrying the same variant. This limitation underscores the importance of combining clinical phenotype, family segregation, and functional data for accurate variant interpretation, particularly for novel missense variants in rare autosomal recessive disorders.

For pediatric patients with congenital cardiac valve disease and progressive cardiomyopathy (even after multiple cardiac surgeries), genetic testing for *PLD1* and other cardiac disease-related genes should be performed early to clarify the genetic etiology. This not only provides a basis for genetic counseling (e.g., prenatal diagnosis for the proband's family) but also helps to develop personalized follow-up and treatment plans to delay the progression of cardiomyopathy.

This study has several limitations. First, we did not perform functional experiments to directly validate the pathogenicity of the two *PLD1* variants—specifically, RNA splicing assays for c.434 + 1G > T or enzymatic activity assays for p.Tyr894Cys—so the mechanistic link remains presumptive and requires confirmation in ongoing work. Second, as a single case report, the causal relationship cannot be definitively established; the observed DCM may partly reflect chronic hemodynamic stress from congenital valve disease and multiple surgeries rather than being solely attributable to *PLD1* deficiency, and the potential roles of genetic modifiers, epigenetic factors, or environmental influences remain unknown, so generalization from this single case warrants caution. Third, although the proband's heterozygous parents were clinically asymptomatic at evaluation, long-term cardiac follow-up is needed to rule out possible subclinical or late-onset manifestations in carriers. Fourth, the second variant (p.Tyr894Cys) remains classified as VUS under the ACMG/AMP framework (4 points: PM3 + PP4 + PM2_Supporting), and reclassification to likely pathogenic would require additional supportive data such as consistent in silico predictions, functional validation, or independent affected cases.

In conclusion, this case reports a patient with CVDD1 and DCM caused by novel compound heterozygous variants in the *PLD1* gene, which enriches the variant spectrum of the *PLD1* gene and provides a basis for genetic diagnosis, treatment, and counseling of such diseases. For patients with unexplained cardiac valve disease and cardiomyopathy, genetic testing should be actively performed to clarify the etiology and provide personalized treatment plans.

## Data Availability

The datasets generated during this study are deposited in publicly accessible repositories. The accession numbers for the variants c.434 + 1G > T and p.Tyr894Cys in the ClinVar database are SCV007579611 and SCV007579610, respectively.
